# Mesenchymal stem cell transplantation improves chronic colitis-associated complications through inhibiting the activity of toll-like receptor-4 in mice

**DOI:** 10.1186/s12876-018-0850-7

**Published:** 2018-08-13

**Authors:** Guo chao Niu, Lei Liu, Libo Zheng, Hong Zhang, David Q. Shih, Xiaolan Zhang

**Affiliations:** 1 Department of Gastroenterology, The Second Hospital of Hebei Medical University, No.80 Huanghe Avenue, Shijiazhuang, Hebei 050035 People’s Republic of China; 2 Hebei Key Laboratory of Gastroenterology, Shijiazhuang, People’s Republic of China; 3 Hebei Institute of Gastroenterology, Shijiazhuang, People’s Republic of China; 4 F. Widjaja Foundation, Inflammatory Bowel & Immunobiology Research Institute, Cedars-Sinai Medical Center, Los Angeles, CA USA

**Keywords:** Chronic colitis, Hepatobiliary complications, Lipopolysaccharide, Mesenchymal stem cells, Toll receptor 4

## Abstract

**Background:**

A variety of extra-intestinal manifestations (EIMs), including hepatobiliary complications, are associated with inflammatory bowel disease (IBD). Mesenchymal stem cells (MSCs) have been shown to play a potential role in the therapy of IBD. This study was designed to investigate the effect and mechanism of MSCs on chronic colitis-associated hepatobiliary complications using mouse chronic colitis models induced by dextran sulfate sodium (DSS).

**Methods:**

DSS-induced mouse chronic colitis models were established and treated with MSCs. Severity of colitis was evaluated by disease activity index (DAI), body weight (BW), colon length and histopathology. Serum lipopolysaccharide (LPS) levels were detected by limulus amebocyte lysate test (LAL-test). Histology and liver function of the mice were checked correspondingly. Serum LPS levels and bacterial translocation of mesenteric lymph nodes (MLN) were detected. Pro-inflammatory cytokines including tumor necrosis factor-α (TNF-α), interferon-γ (IFN-γ), interleukin-1β (IL-1β), interleukin-17A (IL-17A), Toll receptor 4 (TLR4), TNF receptor-associated factor 6 (TRAF6) and nuclear factor kappa B (NF-κB) were detected by immunohistochemical staining, western blot analysis and real-time PCR, respectively.

**Results:**

The DSS-induced chronic colitis model was characterized by reduced BW, high DAI, worsened histologic inflammation, and high levels of LPS and *E. coli*. Liver histopathological lesions, impaired liver function, enhanced proteins and mRNA levels of TNF-α, IFN-γ, IL-1β, IL-17A, TLR4, TRAF6 and NF-κB were observed after DSS administration. MSCs transplantation markedly ameliorated the pathology of colon and liver by reduction of LPS levels and proteins and mRNA expressions of TNF-α, IFN-γ, IL-1β, IL-17A, TLR4, TRAF6 and NF-κB.

**Conclusions:**

MSCs can improve chronic colitis-associated hepatobiliary complications, probably by inhibition of enterogenous endotoxemia and hepatic inflammation through LPS/TLR4 pathway. MSCs may represent a novel therapeutic approach for chronic colitis-associated hepatobiliary complications.

## Background

Inflammatory bowel disease (IBD), characterized by chronic, relapsing immune-mediated gut inflammation, is associated with extraintestinal manifestations (EIMs) involving multiple organs. EIMs are found in 21–47% of IBD patients [1–4]. Higher risk has been also found in IBD patients with hepatobiliary complications than in normal people [5, 6]. They are common parenteral performances, influencing the fatality rate of IBD. The most common EIMs in IBD are hepatobiliary complications, including primary sclerosing cholangitis (PSC), non-alcoholic fatty liver disease (NAFLD), cholelithiasis, primary biliary cirrhosis (PBC), and IgG4-associated cholangitis (IAC). Nevertheless, the pathogenesis of hepatobiliary complications is still unclear. Therefore, it is important to investigate the mechanism of chronic colitis-associated hepatobiliary complications.

The lipopolysaccharide (LPS)/Toll receptor 4 (TLR4) signaling pathway plays a key role in the pathogenesis of chronic liver diseases [7]. LPS, a key component of Gram-negative bacterial cell wall, is released with bacterial clearage or adhere to other cells. TLR4, one of the family members of TLRs, is the main receptors of the innate immunity, identifying pathogens. It is also the surface receptor of LPS, acting with the innate and adaptive immunity systems. As all know, inflammatory cytokines, causing damages to colon and intestinal mucosal barrier, are increased in IBD. It is reported that intestinal endotoxemia (IETM) may be the important pathogenesis mechanism of NAFLD [8]. Animal studies also proved the relevance between bacterial translocation and liver diseases. Some studies showed that LPS also played an important role in the pathogenesis and progress of alcoholic liver damage and fibrosis [9–12].

Mesenchymal stem cells (MSCs) are derived from mesoderm mesenchymal with multiple differentiation potentials to repair various tissue damages. As early as 1998, Lopez et al. [13, 14] found that MSCs transplantation could cure IBD while they were used to treat malignant blood diseases of IBD patients. Other researches showed that MSCs not only accelerated the repair process of radiation-damaged intestinal epithelium of mice [15], but also promoted the proliferation of these cells [16]. The administration of MSCs in mice model of radiation intestinal injury can effectively improve the permeability of the intestinal mucosa and histopathological damages [17]. Systemic application of MSCs was found to be able to repair liver damage, and is able to resist hepatic fibrosis in animal models of chronic liver disease [18, 19]. Protective effects of MSCs have already been reported in different liver injury models [20, 21], and even in patients with autoimmune liver disease [22].

There are few reports about the protective role and mechanism of MSCs in IBD-associated hepatobiliary complications. In this study, a mouse chronic colitis model was established and treated with exogenous infusion human umbilical cord MSCs (hUC-MSCs) to check the protective role and mechanism of MSCs in chronic colitis-associated hepatobiliary complications.

## Methods

### hUC-MSCs

The hUC-MSCs were kindly provided by Alliancells Bioscience Co, Ltd. of Tianjin, China. The hUC-MSCs were isolated and expanded by the company, based on a published method [23, 24]. Flow cytometry was used to analyze and identify the MSC phenotype, as previously described [23]. Briefly, cells were stained with phycoerythrin (PE)-conjugated antibodies against CD11b, CD73, CD90, CD45, CD105 and HLA-DR (all from Santa Cruz Biotechnology, Santa Cruz, CA, USA), and fluorescein isothiocyanate (FITC)-conjugated antibodies against CD19 and CD34 (all from Santa Cruz Biotechnology, Santa Cruz, CA, USA). Mouse isotypic antibodies served as the control. Cells were stained in single label and then analyzed by flow cytometry with a FACS scan (BD Biosciences, Franklin Lake, NJ, USA).

### Animal model

All animal work was approved by the Experimental Animal Center in Hebei Province, (Shijiazhuang, China) and carried out under its procedural and ethical guidelines. Male C57BL/6 J mice (6–8 weeks of age) were randomly allocated to three experimental groups (*n* = 10). Chronic colitis was induced by multiple-cycle administration of dextran sodium sulfate (DSS; 40, 000–50, 000 MW; Sigma) drinking water. Mice received either regular distilled water (control) or 2% (*w*/*v*) DSS drinking water on days 1–5, 8–12, 15–19, 22–26, 29–33, and 36–40. In DSS + MSCs group, 1 × 10^6^/200 μL hUC-MSCs were injected into the caudal vein of each mouse, while the same volume of PBS was given to the control and DSS + vehicle groups. Mice are euthanized by CO2 inhalation followed by cervical dislocation at day 43.

### Biochemical analysis

The serum samples were collected from the femoral artery to subsequently evaluate alanine aminotransferase (ALT; Sigma, St Louis, MO, USA), aspartate aminotransferase (AST; Sigma, St Louis, MO, USA), albumin (ALB; Abcam, Cambridge, United Kingdom), total bilirubin (TBIL; Sigma, St Louis, MO, USA) and direct bilirubin (DBIL; Sigma, St Louis, MO, USA) using commercial kits and strictly according to the manufacturers’ instructions.

### Endotoxin assay

A limulus amebocyte lysate (LAL-Test, GenScript, USA) was used, according to the manufacturer’s instructions.

### Disease activity index (DAI), and macroscopic and histopathological analyses of colonic injury

DAI and colonic damage were assessed blindly according to a standard scoring system, as previously described [25]. Macroscopic inflammation was scored using the established classification. Samples were processed routinely and stained with hematoxylin and eosin (H&E). Histopathological scores of colons were assigned in a blinded manner by two trained animal pathologists. A scoring system that focused on inflammatory cell infiltration and mucosal damage was used, as previously described [24].

### Germiculture of mesenteric lymph node (MLN)

Germiculture of mesenteric lymph nodes (MLN) is an assay of incidence of bacterial translocation. All macroscopically visible MLN were collected under sterile conditions in ice cold Eppendorf tubes and mechanically homogenized in 200 μL of sterile 1× PBS. Then, 0.1 mL of tissue homogenate was cultured on ordinary agar medium at 37 °C for 24 h. Then, the number of bacteria was counted using colony forming-unit (CFU). The rate of bacteria translocation was calculated (germiculture positive specimen number/training specimen total number).

### Histological and immunohistochemistry analysis of liver

Postmortem, the right liver lobe was removed and liver sections were processed routinely. Hematoxylin-eosin (H&E) staining was performed to assess inflammation, and Masson’s trichrome (MT) staining was performed to assess collagen deposition. Liver histopathological scores were assigned independently and in a blind manner by two well-trained pathologists. Inflammation was graded using the histology activity index (HAI-Knodell score) by Knodell [26].

Immunohistochemistry was done following the protocol the Dako protocol (Dako, Glostrup, Denmark). The samples were incubated with mouse polyclonal anti-TNF-α antibody (1:200, Biolegend, San Diego, CA, USA), rat monoclonal anti-IFN-γ antibody (1:100, Biolegend), rabbit polyclonal anti-IL-1β antibody (1:200, Santa Cruz Biotechnology, Santa Cruz, CA, USA), rabbit polyclonal anti-IL-17A antibody (1:200, Santa Cruz), rabbit polyclonal anti-TLR4 antibody (1:200, Santa Cruz), rabbit polyclonal anti-TRAF6 antibody (1:200, Santa Cruz) and mouse polyclonal anti-NF-κB antibody (1:200, Santa Cruz). Peroxidase-conjugated secondary antibody was used with diaminobenzidine (DAB) peroxidase substrate kit (Vector Laboratories, Burlingame, CA, USA). The cells stained brown were considered positive, and the ratio of stained cells to total cells was determine from 10 different fields and calculated using the Image J software (National Institutes of Health, Bethesda, MD, USA).

### Western blot

Western blotting was performed as described previously [27]. Briefly, the tissues were lysed in ice-cold RIPA buffer (150 mM NaCl, 0.1% Triton X-100, 0.5% sodium deoxycholate, 0.1% SDS, 50 mM Tris-HCl pH 8.0 and protease inhibitors) for 2 h at 4 °C, under agitation. Samples were centrifuged at 12,000 rpm for 20 min at 4 °C and the supernatant was decanted. Equal amounts of proteins (50 μg) were separated by 8% polyacrylamide gel electrophoresis (Bio-Rad, Hercules, CA, USA). The proteins were transferred to PVDF membranes (Millipore corp., Billerica, MA, USA) and the membranes were blocked using nonfat powdered milk. The membranes were incubated with the primary antibodies overnight at 4 °C: mouse anti-TNF-α polyclonal (1:500, Biolegend, San Diego, CA, USA), rat anti-IFN-γ monoclonal (1:1000, Biolegend), rat anti-IL-1β polyclonal (1:500, Santa Cruz Biotechnology, Santa Cruz, CA, USA), rat anti-IL-17A polyclonal (1:500, Santa Cruz), rat anti-TLR4 polyclonal (1:500, Santa Cruz), rat anti-TRAF6 polyclonal (1:500, Santa Cruz) and rat anti-NF-κB polyclonal (1:500, Santa Cruz) antibodies. Peroxidase-conjugated secondary antibody was used with diaminobenzidine (DAB) peroxidase substrate kit (Vector Laboratories, Burlingame, CA, USA). The cells stained brown were considered positive, and the ratio of stained cells to total cells was determine from 10 different fields and calculated using the Image J software (National Institutes of Health, Bethesda, MD, USA).

### Real-time PCR

Total RNA was isolated and purified from liver samples using TRIpure Reagent (Aidlab Biotechnologies Co. Ltd., China). cDNA synthesis and real-time PCR were performed as described before [26]. Primers were purchased from Saibaisheng Gene Co. Ltd., China. The primers were designed as: TNF-α-forward 5′-GGA AAG GAC GGA CTG GTG TA-3′, TNF-α-reverse 5’-TGC CAC TGG TCT GTA ATC CA-3′; IFN-γ-forward 5’-GGC AAG TTC AAC GGC ACA G-3′, IFN-γ-reverse 5’-CGC CAG T AG ACT CCA CGA CAT-3′; IL-1β-forward 5′-G AGC ACC TTC TTT TCC TTC ATC TT-3′, IL-1β-reverse 5’-TCA CAC ACC AGC AGG TTA TCA TC-3′; IL-17A-forward 5′-GGA AAG GAC GGA CTG GTG TA-3′, IL-17A-reverse 5′-TG C CAC TGG TCT GTA ATC CA-3′; TLR4-forward 5′-TTT ATT CAG AGC CGT TGG- 3′ TLR4-reverse 5’-CCC ATT CCA GGT AGG TGT-3′; TRAF6-forward 5′-GTA TC C GCA TTG AGA AGC-3′, TRAF6-reverse 5’-GCA GTG AAC CAT CCG TGT-3′; NF-κB-forward 5′-AAG GAT TCG AGC AGT TAG-3′, NF-κB-reverse 5′-AAG AGT TGG TGA TAG GCT-3′; GAPDH-forward 5’-GGC AAG TTC AAC GGC ACA G-3′, and GAPDH-reverse 5’-CGC CAG TAG ACT CCA CGA CAT-3′. Data were analyzed using the 2^-ΔΔCt^ method [28].

### Statistical analysis

Data were expressed as means ± standard deviation (mean ± SD) and analyzed with SPSS 13.0 (SPSS Inc., Chicago, IL, USA) by one-way ANOVA and the SNK post hoc test. *P* < 0.05 was considered significant.

## Results

### Isolation and identification of hUC-MSC

**Fig. 1:**
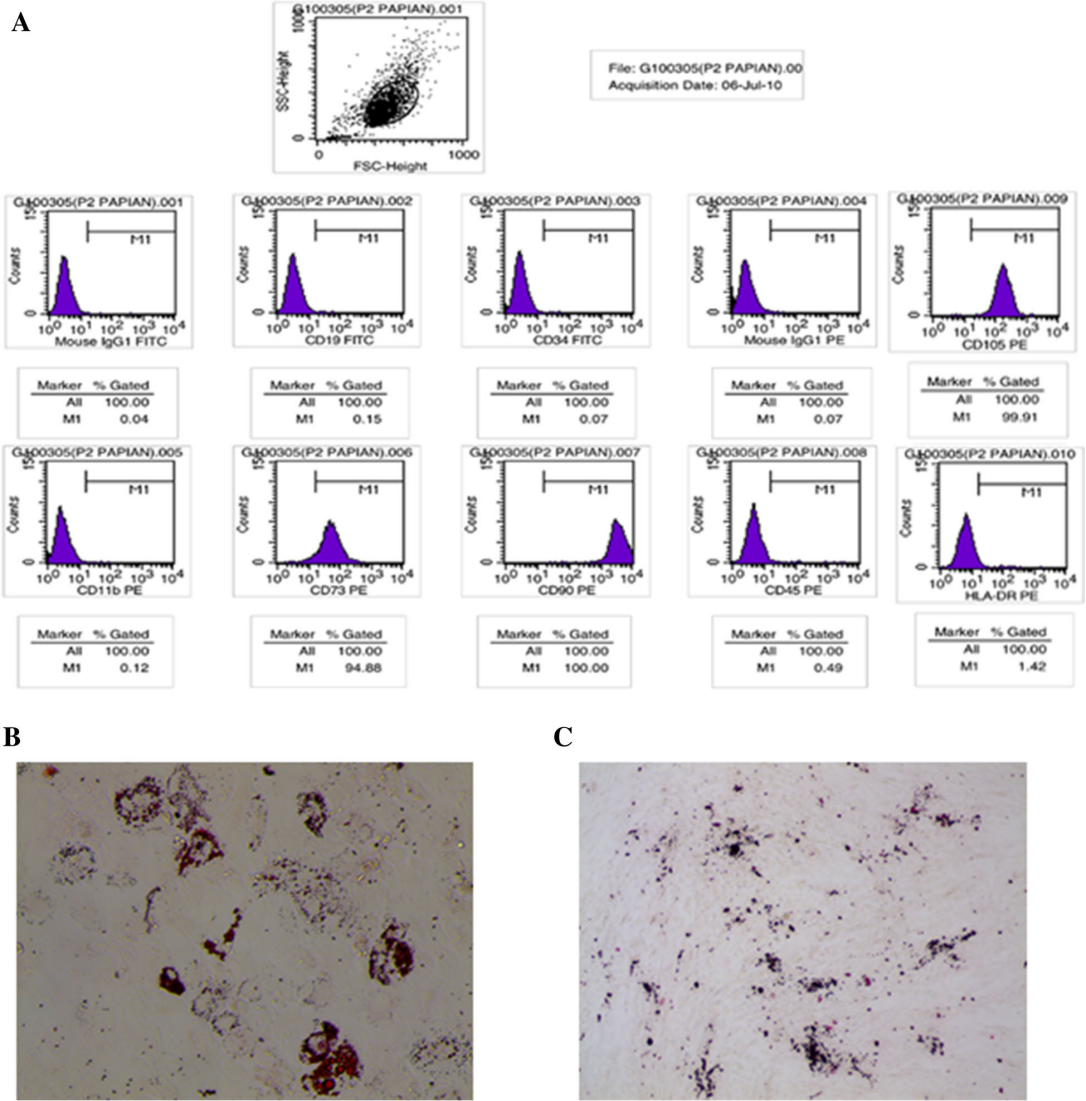
Characterization of UC-MSCs. **a** Flow cytometric analysis of cell surface markers on UC-MSCs. **b** adipogenesis differentiation of UCMSCs was analyzed by Oil-Red-O staining. **c** Osteogenesis differentiation was analyzed by Alizarin Red-S staining. US-MSC, umbilical cordderived mesenchymal stem cell

The hUC-MSCs wereisolated from umbilical cords and pooled from a group of four individual donors. The cells were cultured and expanded in vitro in the ultimate conditions. At the end of culture, flow cytometric analysis was performed with a panel of surface markers, which confirmed that the cells were positive for CD73, CD90 and CD105, and negative for CD11b, CD45, HLA-DR, CD19 and CD34 (Fig. 1a). To further confirm the multipotentiality of UC-MSCs after in vitro culture, we assessed their abilities to differentiate into cells of osteogenic and adipogenic lineages. As previously described, UC-MSCs were cultured in the appropriate inducing media for 2 or 3 weeks, and the lipid vacuoles were stained with Oil Red O in the differentiated adipocytes (Fig. 1b), whereas the osteogenic differentiation of MSCs was stained with AlizarinRed (Fig. 1c). These data demonstrate that the UC-MSCs maintained their stem cell characteristics after in vitro culture and expansion.

### MSCs improved the clinical symptoms of established chronic colitis models

**Fig. 2:**
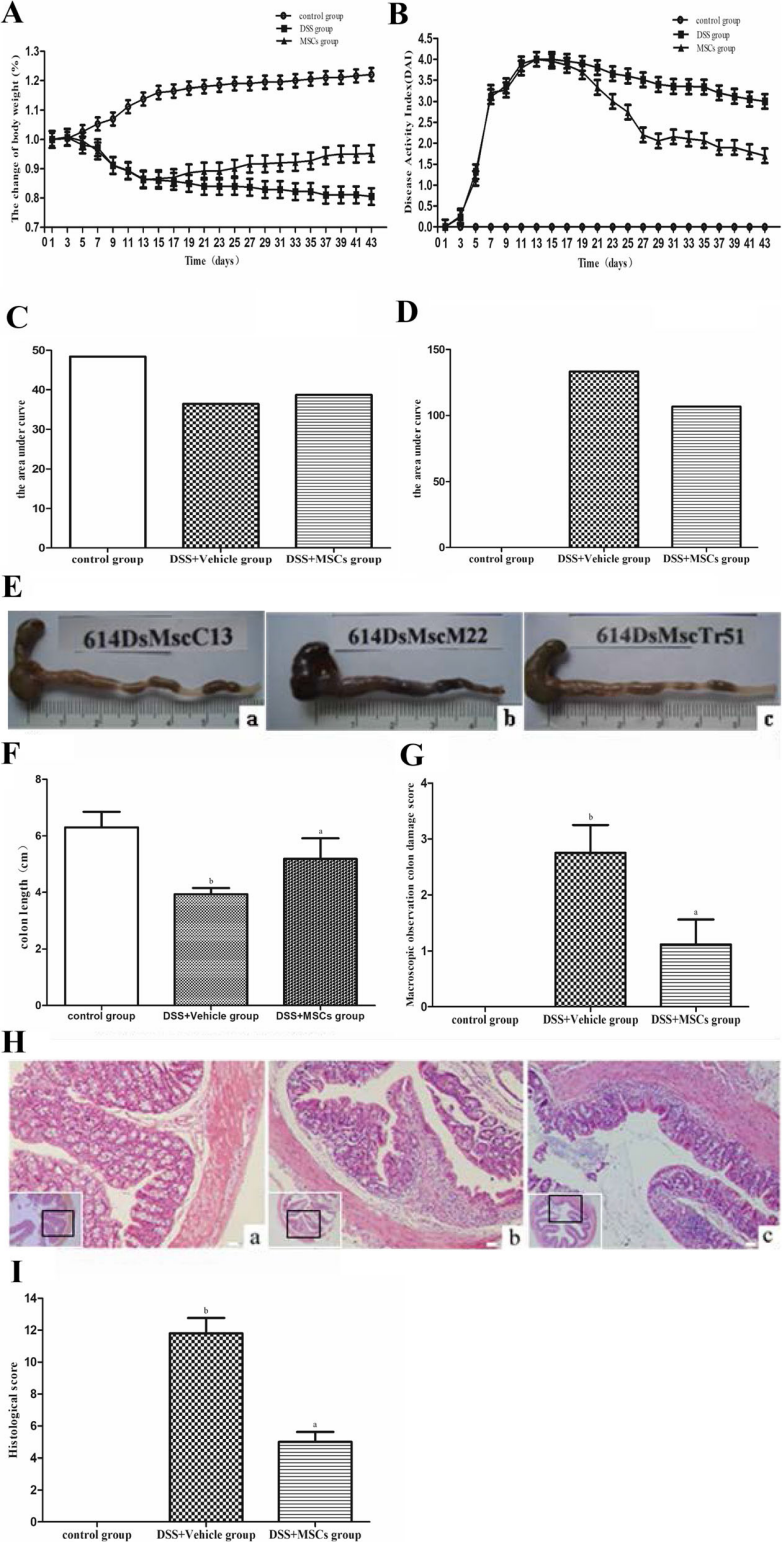
The chronic DSS-induced colitis model was established. **a**, **c** Body weight (BW) was assessed daily and expressed as percentage of baseline BW. **b**, **d** Disease activity index (DAI), consisting of BW loss, stool consistency and OB, was measured daily. The DSS + vehicle group (**b**) showed more severe inflammation in the colon compared to the control group **a**, while the inflammation in the colon in the DSS + MSCs group was ameliorated. **e** Samples of colon. **f** Colon length. **g** Morphology score. **h** H&E staining (× 200). **i** Histological score (^a^*P* < 0.05 vs. the DSS + vehicle group; ^b^*P* < 0.05 vs. the control group). Data were expressed as mean ± SD

Compared with the control group, mice in the DSS + vehicle group all developed consistent clinical signs of colitis after DSS administration, including significant loss of BW (22.5% ± 1.90% vs. 0.00% ± 0.00, *P* < 0.05) (Fig. 2a, c), stool blood, lassitude, and increased DAI (4.00 ± 0.65 vs. 0.00 ± 0.00, *P* < 0.05) (Fig. 2b, d). MSCs infusion significantly reduced the extent of loss of BW (4.30% ± 1.38% vs. 14.6% ± 1.18%, *P* < 0.05) and DAI as well (0.30 ± 0.46 vs. 3.05 ± 0.25, *P* < 0.05) compared to the DSS + vehicle group.

### MSCs inhibited colonic inflammation

Increased macroscopic gut inflammation was observed in the DSS + vehicle group compared to the control group (Fig. 2e). Shorter colon length and more severe inflammation were also observed in the DSS + vehicle mice compared to the control group (3.93 ± 0.22 vs. 6.30 ± 0.55, *P* < 0.05), which were improved by MSCs administration (5.18 ± 0.73 vs. 3.93 ± 0.22, *P* < 0.05) (Fig. 2f). In addition, the degrees of congestion, edema of the colon wall and infiltration of inflammatory cell into the mucosa in the DSS + vehicle group were significantly increased compared with the control group (2.75 ± 0.50 vs. 0.00 ± 0.00, *P* < 0.05), which were reduced by MSCs compared to the DSS + vehicle group (1.11 ± 0.45 vs. 2.75 ± 0.50, *P* < 0.05) (Fig. 2g). Histological score of the DSS + vehicle group was significantly higher than that of the control group (11.80 ± 0.96 vs. 0.00 ± 0.00, *P* < 0.05), which was significantly lowered in the DSS + MSCs group (5.00 ± 0.62 vs. 11.80 ± 0.96, *P* < 0.05) (Fig. 2h, i).

### MSCs attenuated the DSS-induced hepatobiliary inflammation

**Fig. 3:**
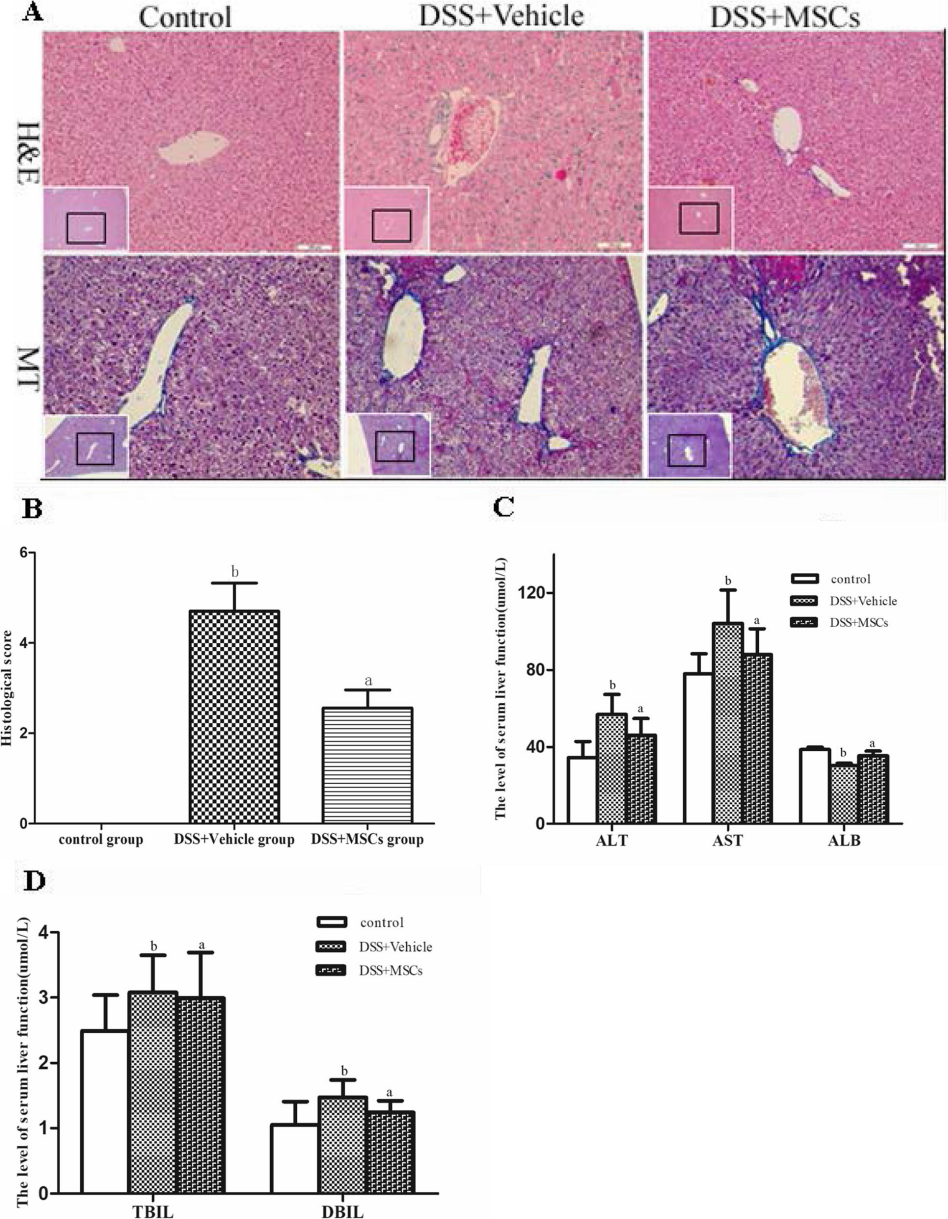
MSCs alleviated chronic colitis-associated hepatobiliary disorders. The DSS + vehicle group showed more severe hepatobiliary disorders compared to that of the control group, while MSCs ameliorated hepatobiliary disorders associated with chronic colitis. **a** Representative sections samples of liver (H&E and MT staining; original magnifications, × 200). **b** Histological score (^a^*P* < 0.05 vs. the DSS + vehicle group; ^b^*P* < 0.05 vs. the control group). **c**, **d** Liver function, ALT and AST levels in the DSS + vehicle group were increased and ALB level were lowered compared to that of the control group. ALT and AST levels in the DSS + MSCs group were significantly lower and ALB levels were increased (^a^*P* < 0.05 vs. the DSS + vehicle group; ^b^*P* < 0.05 vs. the control group). TBIL and DBIL levels were not changed (^a^*P* > 0.05 vs. the DSS + vehicle group; ^b^*P* > 0.05 vs. the control group). Data were expressed as mean ± SD

H&E staining and MT staining were performed to evaluate the effect of MSCs administration on liver pathology (Fig. 3a). Cholangitis, as shown by proliferating bile duct, bile thrombus, necrosis and infiltrated inflammatory cells in sinusoids and centrilobular regions, was found in the DSS + vehicle group. At the same time point, extensive histomorphological signs of chronic hepatitis in DSS mice was observed. MT staining revealed that mice exposed to DSS displayed non-significant perisinusoidal fibrosis, and the degree of collagen deposition did not differ between groups, but differences in inflammatory infiltration were detected. The hepatic histological injury was mainly seen in the central vein and portal area. After MSCs transplantation, histological analyses of liver tissue sections revealed that DSS + MSCs mice appeared to be less alleviated than the DSS + vehicle group, indicating the decreasing inflammatory process. Higher score was found in the DSS + vehicle group compared with the control group (4.70 ± 0.62 vs. 0.00 ± 0.00, *P* < 0.05), based on the HAI-Knodell score system, which was decreased by MSCs treatment (2.56 ± 0.40 vs. 4.70 ± 0.62, *P* < 0.05) (Fig. 3b).

### MSCs improved the liver function

Mice in the DSS + vehicle group showed significantly higher levels of ALT and AST (56.82 ± 10.40 vs. 34.50 ± 8.23, *P* < 0.05; 104.25 ± 17.26 vs. 78.00 ± 10.40, *P* < 0.05), and lower levels of ALB than the control group (30.33 ± 1.21 vs. 38.60 ± 1.18, *P* < 0.05). Treatment with MSCs can improve the liver function by reduction of ALT and AST (46.00 ± 8.70 vs. 56.82 ± 10.40, *P* < 0.05; 87.94 ± 13.51 vs. 104.25 ± 17.26, *P* < 0.05) and increase of ALB compared to the DSS + vehicle group (35.43 ± 2.43 vs. 30.33 ± 1.21, *P* < 0.05) (Fig. 3c, d).

### MSCs reduced the bacterial translocation rate and serum LPS levels

**Fig. 4:**
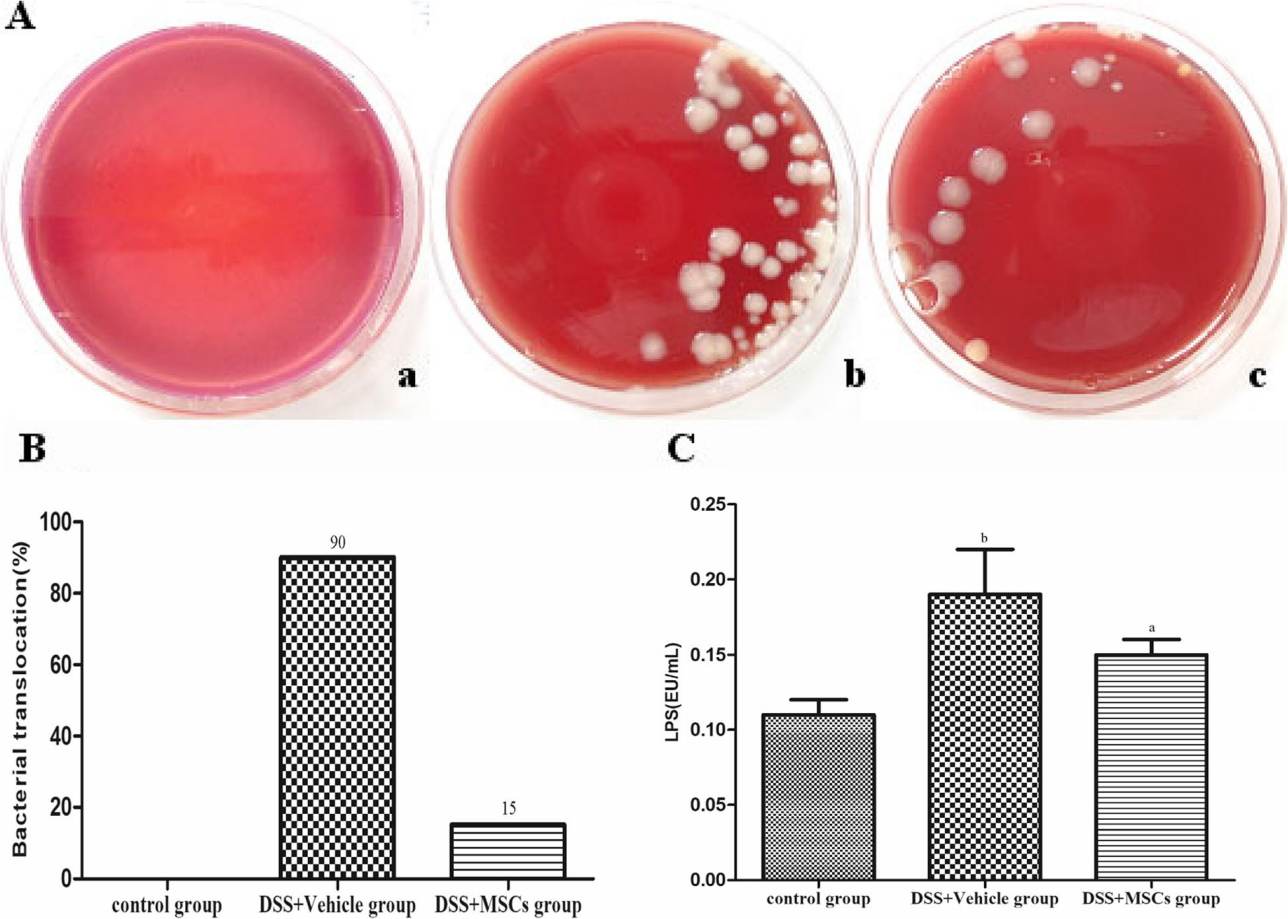
Effect of MSCs on intestinal permeability. (**A**) Bacteria cultured from MLN. Viable bacteria are mainly *Escherichia coli* in the control group (a), DSS + vehicle group (b), and DSS + MSCs group (c). (**B**) Differences in incidence of bacterial translocation to MLN were displayed, Bacterial translocation was increased in the DSS + vehicle group (90%) compared with that of the control group (0%), while bacterial translocation to mesenteric lymph nodes decreased in the DSS + MSCs group (15%). (**C**) The changes of LPS levels. LPS level in the DSS + vehicle group were increased obviously compared with the control group. In the DSS + MSCs group, the LPS levels were lowered compared with that of the DSS + vehicle group. Data were expressed as mean ± SD. ^a^*P* < 0.05 vs. the DSS + vehicle group; ^b^*P* < 0.05 vs. the control group

Bacterial translocation can be used for measuring intestinal permeability. Large amounts of *E. coli* were observed in MLN homogenates from the DSS + vehicle group compared to the control group (bacterial translocation rate, 90% vs. 0%). MSCs significantly reduced bacteria translocation to MLN in the DSS + MSCs group compared to the DSS + vehicle group (bacterial translocation rate, 15% vs. 90%) (Fig. 4a, b). Consistent with bacterial translocation, mice in the DSS + vehicle group showed significantly increased level of LPS compared with the control group (0.19 ± 0.03 vs. 0.11 ± 0.01, *P* < 0.05), which was significantly decreased by MSCs treatment (0.15 ± 0.01 vs. 0.19 ± 0.03, *P* < 0.05) (Fig. 4c).

### MSCs suppressed the mRNA and protein expressions of TNF-α, IFN-γ, IL-1β and IL-17A

**Fig. 5:**
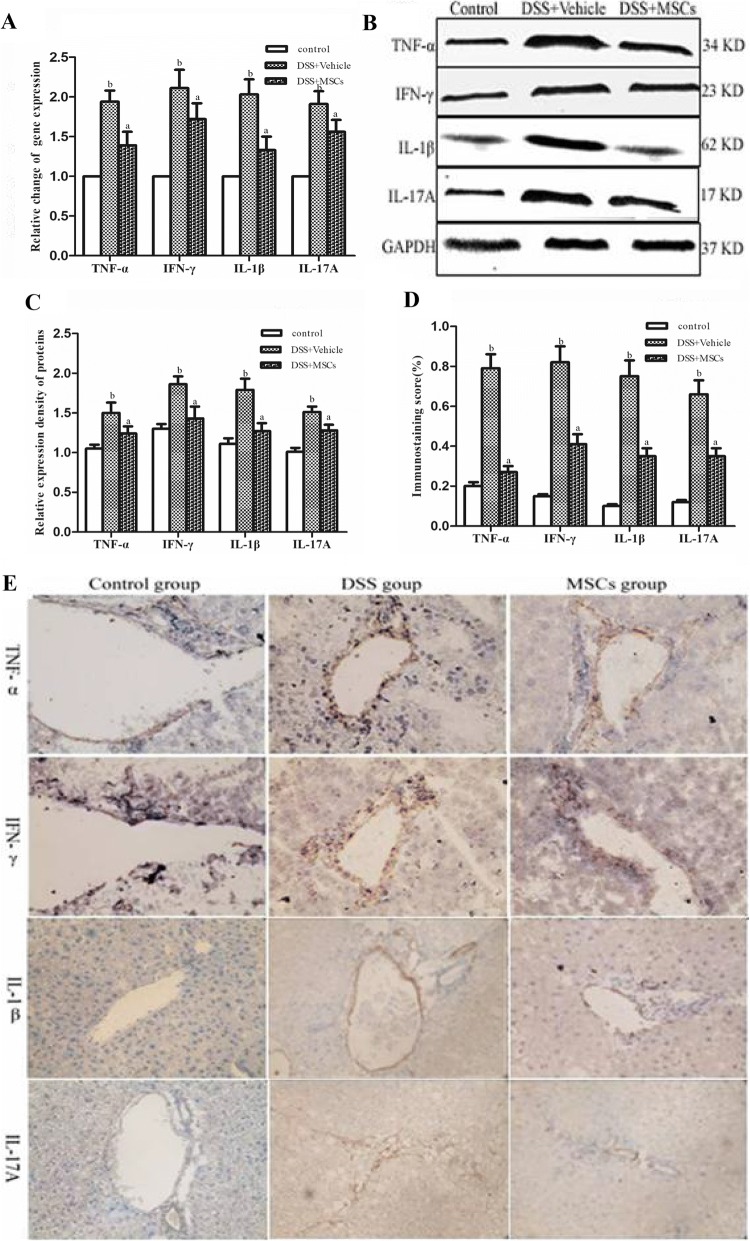
MSCs suppressed the expressions of TNF-α, IFN-γ, IL-1β and IL-17A mRNA and proteins in the liver tissues of chronic DSS-induced colitis. **a** MSCs changed the expressions of TNF-α, IFN-γ, IL-1β and IL-17A mRNA in liver tissues of chronic colitis-associated hepatobiliary disorders. Data were expressed as mean ± SD. ^a^*P* < 0.05 vs. the DSS + vehicle group; ^b^*P* < 0.05 vs. the control group. **b** Representative western blots showing TNF-α, IFN-γ, IL-1β and IL-17A proteins expressions. **c** Expressions of TNF-α, IFN-γ, IL-1β and IL-17A staining in the DSS + vehicle group were higher than that of the control group, while in the DSS + MSCs group, these expressions decreased. **d** The expressions of the TNF-α, IFN-γ, IL-1β, and IL-17A protein in liver tissues were significantly increased in the DSS + vehicle group compared with that of the control group, while they were decreased in the DSS + MSCs group. **e** Quantification of cytokines by immunohistochemistry (original magnifications, × 400)

TNF-α, IFN-γ, IL-1β and IL-17A were expressed by inflammatory cells close to bile ducts area in inflamed portal tracts, and distributed as spotty or diffuse yellow or brown particles in the cell membrane and cytoplasm. We determined the mRNA expressions of TNF-α, IFN-γ, IL-1β and IL-17A using real-time PCR, finding significantly increased mRNA expression in the DSS + vehicle group (1.94 ± 0.14 vs. 1.00 ± 0.00, *P* < 0.05; 2.11 ± 0.23 vs. 1.00 ± 0.00, *P* < 0.05; 2.03 ± 0.19 vs. 1.00 ± 0.00, *P* < 0.05; 1.91 ± 0.16 vs. 1.00 ± 0.00, *P* < 0.05) compared to the control group, and all of them were significantly reduced by MSCs administration (1.39 ± 0.17 vs. 1.94 ± 0.14, *P* < 0.05; 1.72 ± 0.20 vs. 2.11 ± 0.23, *P* < 0.05; 1.33 ± 0.17 vs. 2.03 ± 0.19, *P* < 0.05; 1.56 ± 0.15 vs. 1.91 ± 0.16, *P* < 0.05) (Fig. 5a).

**Fig. 6:**
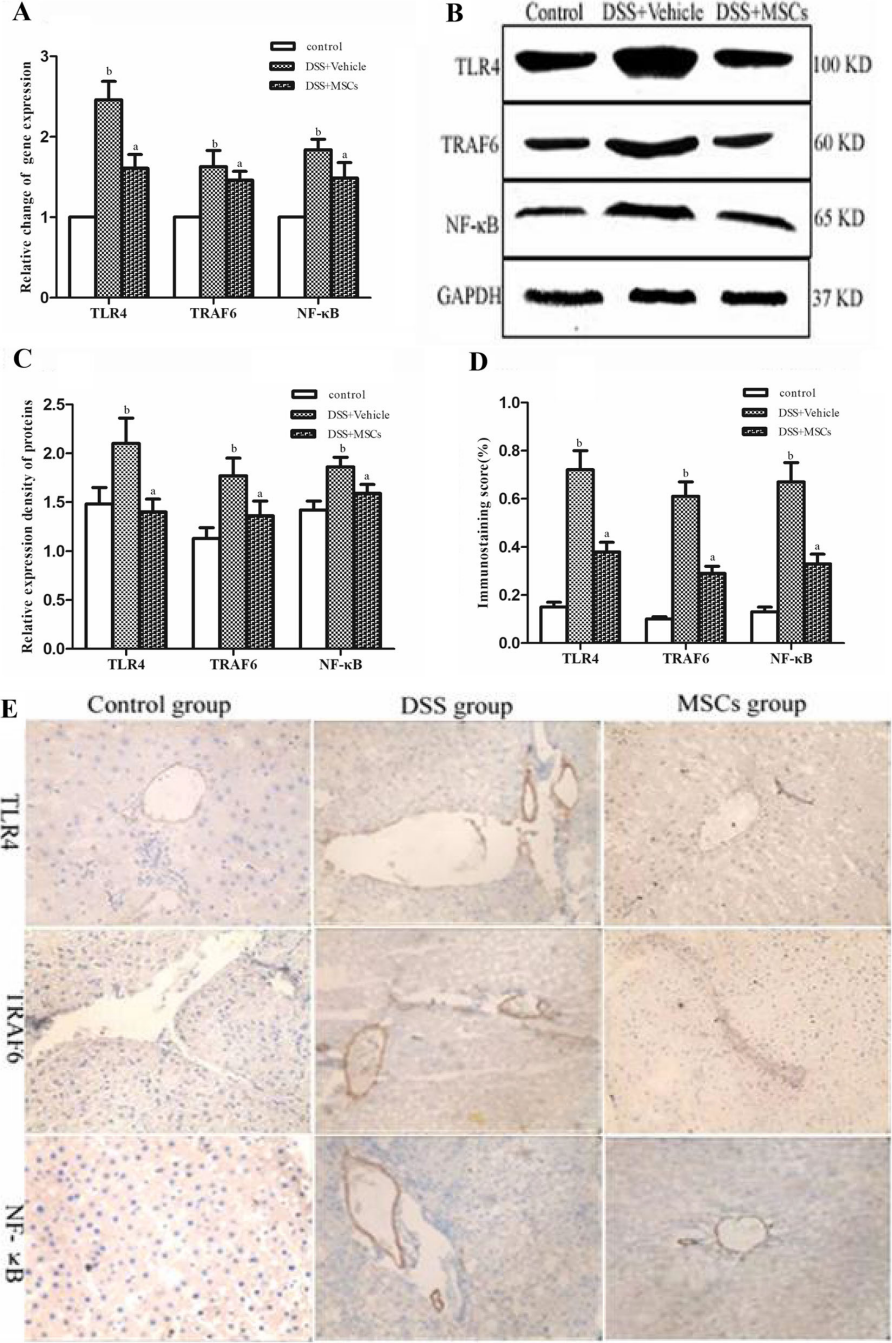
MSCs suppressed the expressions of TLR4, TRAF6 and NF-κB mRNA and proteins in the liver tissues of chronic DSS-induced colitis. **a** MSCs changed the expressions of TLR4, TRAF6 and NF-κB mRNA in liver tissues of chronic colitis-associated hepatobiliary disorders. Data were expressed as mean ± SD. ^a^*P* < 0.05 vs. the DSS + vehicle group; ^b^*P* < 0.05 vs. the control group. **b** Representative western blots showing TLR4, TRAF6 and NF-κB protein expressions. **c** Expressions of TLR4, TRAF6 and NF-κB staining in the DSS + vehicle group were higher than that of the control group, while in the DSS + MSCs group, these expressions decreased. **d** The expressions of the TNF-α, IFN-γ, IL-1β and IL-17A proteins in liver tissues were increased in the DSS + vehicle group compared with that of the control group, while they were decreased in the DSS + MSCs group. **e** Quantification of cytokines by image analysis of immunohistochemistry (original magnifications, × 400)

We next determined the expressions of TNF-α, IFN-γ, IL-1β and IL-17A using western blot. Compared to the control group, expressions of TNF-α, IFN-γ, IL-1β and IL-17A were increased in the DSS + vehicle group (0.79 ± 0.07 vs. 0.20 ± 0.02, *P* < 0.05; 0.82 ± 0.08 vs. 0.15 ± 0.01, *P* < 0.05; 0.75 ± 0.08 vs. 0.10 ± 0.01, *P* < 0.05; 0.66 ± 0.07 vs. 0.12 ± 0.01, *P* < 0.05) (Fig. 6b, c), but all of them were reduced by MSCs administration (0.27 ± 0.03 vs. 0.79 ± 0.08, *P* < 0.05; 0.41 ± 0.05 vs. 0.82 ± 0.08, *P* < 0.05; 0.35 ± 0.04 vs. 0.75 ± 0.08, *P* < 0.05; 0.35 ± 0.04 vs. 0.66 ± 0.07, *P* < 0.05) (Fig. 5a, b). Relative expressions of TNF-α, IFN-γ, IL-1β and IL-17A proteins by western blot were all significantly higher in the DSS + vehicle group compared to the control group (1.50 ± 0.13 vs. 1.05 ± 0.05, *P* < 0.05; 1.86 ± 0.10 vs. 1.30 ± 0.06, *P* < 0.05; 1.79 ± 0.14 vs. 1.11 ± 0.07, *P* < 0.05; 1.51 ± 0.07 vs. 1.01 ± 0.05, *P* < 0.05) (Fig. 5c, d), and all of them were decreased by MSCs administration (1.24 ± 0.09 vs. 1.50 ± 0.13, *P* < 0.05; 1.43 ± 0.15 vs. 1.86 ± 0.10, *P* < 0.05; 1.27 ± 0.10 vs. 1.79 ± 0.14, *P* < 0.05; 1.28 ± 0.07 vs. 1.51 ± 0.07, *P* < 0.05) (Fig. 5d, e).

### MSCs inhibited mRNA and protein expressions of TLR4, TRAF6 and NF-κB

Compared to the control group, relative expressions of TLR4, TRAF6 and NF-κB mRNA of liver tissue were increased in the DSS + vehicle group compared to the control group (2.46 ± 0.23 vs. 1.00 ± 0.00, *P* < 0.05; 1.63 ± 0.20 vs. 1.00 ± 0.00, *P* < 0.05; 1.84 ± 0.13 vs. 1.00 ± 0.00, *P* < 0.05), and all of them were reduced by MSCs administration (1.61 ± 0.17 vs. 2.46 ± 0.23, *P* < 0.05; 1.46 ± 0.11 vs. 1.63 ± 0.20, *P* < 0.05; 1.49 ± 0.19 vs. 1.84 ± 0.13, *P* < 0.05) (Fig. 6a). Relative expressions of TLR4, TRAF6 and NF-κB proteins from western blot were all significantly higher in the DSS + vehicle group compared to the control group (2.10 ± 0.26 vs. 1.48 ± 0.17, *P* < 0.05; 1.77 ± 0.18 vs. 1.13 ± 0.11, *P* < 0.05; 1.86 ± 0.10 vs. 1.42 ± 0.09, *P* < 0.05) (Fig. 6b, c), and all of them were lowered by MSCs administration (1.40 ± 0.13 vs. 2.10 ± 0.26, *P* < 0.05; 1.36 ± 0.15 vs. 1.77 ± 0.18, *P* < 0.05; 1.59 ± 0.09 vs. 1.86 ± 0.10, *P* < 0.05). Positive expressions of TLR4, TRAF6 and NF-κB by immunohistochemistry in the DSS + vehicle group were much higher compared with the control group (0.72 ± 0.08 vs. 0.15 ± 0.02, *P* < 0.05; 0.61 ± 0.06 vs. 0.10 ± 0.01, *P* < 0.05; 0.67 ± 0.08 vs. 0.13 ± 0.02, *P* < 0.05) (Fig. 6d, e), and all of them were reduced by MSCs administration(0.38 ± 0.04 vs. 0.72 ± 0.08, *P* < 0.05; 0.29 ± 0.03 vs. 0.61 ± 0.06, *P* < 0.05; 0.33 ± 0.04 vs. 0.67 ± 0.08, *P* < 0.05).

### Correlation analysis

**Fig. 7:**
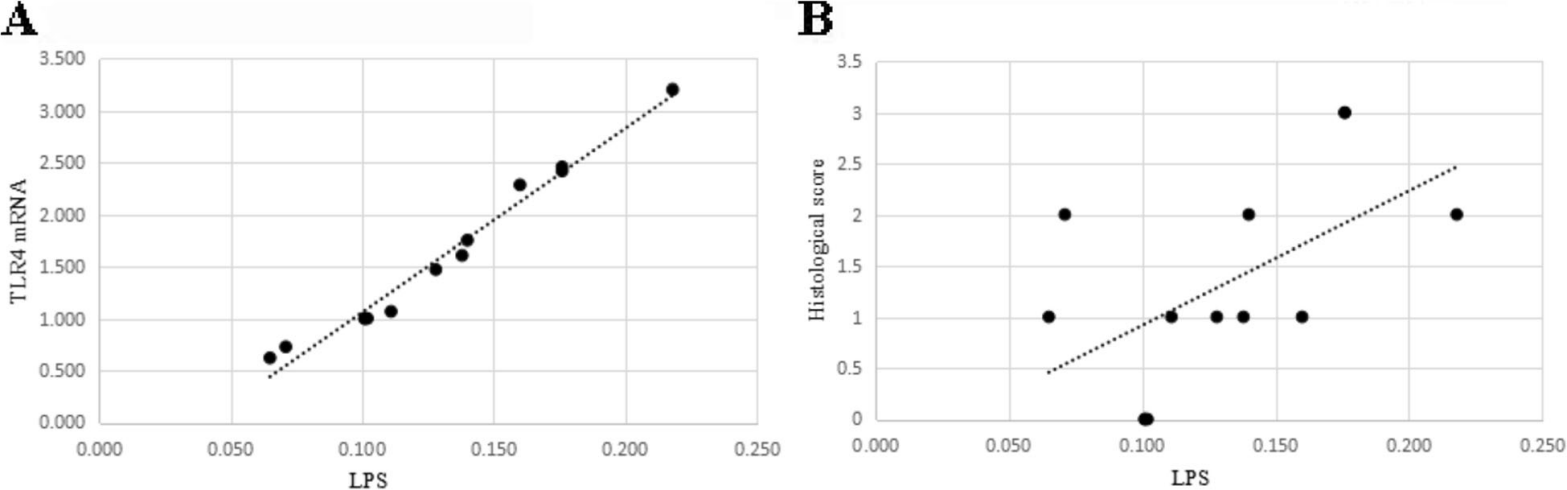
Serum LPS levels depicted positive correlation with TLR4 mRNA expression in liver tissues and HAI-Knodell score. Pearson’s correlation analysis between LPS and TLR4Mrna **a**, LPS and histological score (**b**) were done

Pearson’s correlation analysis showed that serum LPS levels depicted positive correlation with TLR4 mRNA expression in liver tissues and HAI-Knodell score (Fig. 7a, b). The *r* values were 0.901 and 0.825 respectively, *P* < 0.05.

## Discussion

IBD is characterized by a chronic inflammation of the gastrointestinal tract associated with several EIMs. One the EIMs is hepatobiliary complications, which are described as: 1) diseases may share common pathogenic mechanisms with IBD, including PSC, small-duct PSC/pericholangitis and PSC/autoimmune hepatitis overlap; 2) diseases that reflect the degree of pathophysiological damage seen with IBD, including cholelithiasis and portal vein thrombosis; 3) diseases that are related to adverse effects from the treatment of IBD; and 4) diseases that have unknown pathogenesis mechanisms, including IAC, PBC, non-alcoholic fatty liver disease, granulomatous hepatitis, and amyloidosis [29].

In 1998, Marshall et al [30] proposed the “gut-liver axis” showing that intestine and liver can be influenced by each other. Recently, more studies have focused on IBD-associated hepatobiliary complications. Yang et al. [5] found that 35.97% of 606 IBD patients had cholelithiasis, and 68.81% of them had NAFLD. Solis et al [8] also found hepatobiliary complications in IBD patients, higher risk in which was showed in Parente’s study [6]. Zhang et al [31] found necrosis of hepatic cells in liver tissues of mice CD model, and also found lymphocytic infiltration around the central vein and central intralobular in this model without bile duct damage. Gabele et al [12] also showed that DSS-induced colitis enhanced hepatic inflammation and fibrogenesis in experimental NASH through the “gut-liver axis”. Depending on previous publications, DSS-induced chronic colitis model was established in this study to investigate colitis-associated hepatobiliary complications.

DSS administration caused increased levels of serum ALT and AST, and decreased ALB. Examination of liver sections from the mice receiving DSS showed histological signs of cholangitis and chronic hepatitis, similar to non-specific early PSC and AIH, such as hepatocyte ballooning, necrosis, inflammatory cells infiltration, bile duct proliferation, and few bile thrombus. The liver injury was alleviated by treatment with MSCs, as reflected by decreased levels of ALT and AST and increased ALB. Concurrently, histological signs of liver damage appeared to be less severe after MSCs treatment. Thus, was there any relationship between chronic colitis-associated hepatobiliary complications and colonic inflammation?

The liver is vulnerable to exposure to bacterial products translocated from the gut lumen via the portal vein because of the “gut-liver axis”, in particular during disruption of the intestinal epithelial barrier [32–34]. One of the components in the cell wall of Gram-negative bacteria is LPS. Generally, only a small amount of LPS can be translocated into portal blood reaching the liver [35]. Experimental chronic liver damage is commonly associated with increased bacterial translocation [36, 37]. There are increasing evidence that gut-derived microbial components represent danger signals for the host in the liver, inducing the inflammatory cascade activation of immune cells and modulating functions and responses of liver parenchymal cells [38]. Our data demonstrated that mice receiving DSS had impaired intestinal mucosa by increased bacterial translocation rate with high serum level of LPS as well. The striking suppression of LPS levels by MSCs was accompanied with reduced bacterial translocation rate.

Toll-like receptors (TLRs), expressed on Kupffer cells, endothelial cells, dendritic cells, biliary epithelial cells, hepatic stellate cells, and hepatocytes, can be activated by gut-derived bacterial products, causing the activation of these cells in acute and chronic liver injuries [39]. One of the TLRs proteins, TLR4, is a membrane pattern recognition receptor, playing a key role in endotoxin-induced innate immune system activation [40, 41]. The TLR4 signaling transduction pathway consists in the activation of various transcription factors such as NF-κB via affecting downstream of TRAF6, which induces directly or indirectly pro-inflammatory cytokines expressions in the KC: TNF-α, IL-1β, IFN-γ and IL-17A. These cytokines will further increase pro-inflammatory cytokines and chemoattractant factors production in the liver, leading to the attraction of other immune cells, such as neutrophils, monocytes and lymphocytes [35, 42, 43]. The liver inflammatory cascade in our study was probably triggered by the gut-derived LPS through a TLR4-dependent mechanism, as described above.

This study showed that a large number of *E. coli* cells grew from cultured MLN from the mice receiving DSS, and the serum level of LPS was significantly increased with increased expressions of TLR4, TRAF6, and NF-κB protein and mRNA. Nevertheless, both of them could be decreased by MSCs treatment. Serum LPS levels were positively correlated with TLR4 mRNA expression. Data also showed that TNF-α, IFN-γ, IL-1β and IL-17A proteins and mRNA expressions were increased from the mice receiving DSS and decreased by MSCs treatment. Correlation analysis showed that serum LPS levels were positively correlated with the HAI-Knodell score, revealing that endotoxemia promoted the release of the above inflammatory mediators, which can cause hepatobiliary complications. Hence, this data suggested that increased LPS could aggravate liver injury, probably through the inhibition of TLR4 pathway during the process of IBD.

Of course, the present study has some limitations. Our future studies will examine the role of tight junctions and adhesion molecules in the intestine in this mouse model of colitis.

## Conclusions

In conclusion, those data supported that MSCs treatment could effectively alleviate intestinal inflammation, reduce mucosal permeability, and improve IETM and hepatobiliary complications through LPS/TLR4. This may provide a new targeted therapy of IBD-associated hepatobiliary complications.

## Abbreviations

ALB: Albumin
ALT: Alanine aminotransferase
AST: Aspartate aminotransferase
BW: Body weight
CFU: Colony forming-unit
DAI: Disease activity index
DBIL: Direct bilirubin
DSS: Dextran sulfate sodium
EIMs: Extra-intestinal manifestations
H&E: Hematoxylin and eosin
hUC-MSCs: Human umbilical cord MSCs
IAC: IgG4-Associated cholangitis
IBD: Inflammatory bowel disease
IETM: Intestinal endotoxemia
IFN-γ: Interferon-γ
IL-17A: Interleukin-17A
IL-1β: Interleukin-1β
LAL: Limulus amebocyte lysate
LPS: Lipopolysaccharide
MLN: Mesenteric lymph nodes
MSCs: Mesenchymal stem cells
MT: Masson’s trichrome
NAFLD: Non-alcoholic fatty liver disease
NF-κB: Nuclear factor kappa B
PBC: Primary biliary cirrhosis
PSC: Primary sclerosing cholangitis
TBIL: Total bilirubin
TLR4: Toll receptor 4
TLRs: Toll-like receptors
TNF-α: Tumor necrosis factor-α
TRAF6: TNF receptor-associated factor 6
